# The influence of vocal training and acting experience on measures of voice quality and emotional genuineness

**DOI:** 10.3389/fpsyg.2014.00156

**Published:** 2014-03-07

**Authors:** Steven R. Livingstone, Deanna H. Choi, Frank A. Russo

**Affiliations:** ^1^Department of Psychology, Ryerson UniversityToronto, ON, Canada; ^2^Toronto Rehabilitation InstituteToronto, ON, Canada; ^3^Department of Psychology, Queen’s UniversityKingston, ON, Canada

**Keywords:** singing, emotion, emotional genuineness, acting, training, individual differences, voice quality, linear mixed models

## Abstract

Vocal training through singing and acting lessons is known to modify acoustic parameters of the voice. While the effects of singing training have been well documented, the role of acting experience on the singing voice remains unclear. In two experiments, we used linear mixed models to examine the relationships between the relative amounts of acting and singing experience on the acoustics and perception of the male singing voice. In Experiment 1, 12 male vocalists were recorded while singing with five different emotions, each with two intensities. Acoustic measures of pitch accuracy, jitter, and harmonics-to-noise ratio (HNR) were examined. Decreased pitch accuracy and increased jitter, indicative of a lower “voice quality,” were associated with more years of acting experience, while increased pitch accuracy was associated with more years of singing lessons. We hypothesized that the acoustic deviations exhibited by more experienced actors was an intentional technique to increase the genuineness or truthfulness of their emotional expressions. In Experiment 2, listeners rated vocalists’ emotional genuineness. Vocalists with more years of acting experience were rated as more genuine than vocalists with less acting experience. No relationship was reported for singing training. Increased genuineness was associated with decreased pitch accuracy, increased jitter, and a higher HNR. These effects may represent a shifting of priorities by male vocalists with acting experience to emphasize emotional genuineness over pitch accuracy or voice quality in their singing performances.

The goals of a singer are varied and many: accurate pitch reproduction, desired voice quality, clear intelligibility, precise timing, and intended emotional inflection; these factors are not independent, and how they are prioritized may reflect differences in the training and experience of a performer (Ostwald, 2005; Bunch, 2009). Two types of training that may differentially affect vocal acoustic goals are singing training and acting experience. Numerous studies have investigated the acoustics of the expert singing voice (Sundberg, 2003), and the effects of short-term training on singing acoustics (Smith, 1963; Brown et al., 2000; Awan and Ensslen, 2010). The acoustic qualities of the trained actor’s speaking voice have also been studied, though less extensively (Nawka et al., 1997; Bele, 2006), as have the effects of short-term acting training on speech acoustics (Timmermans et al., 2005; Walzak et al., 2008). To the authors’ knowledge, there has only been one study that has considered the influence of acting training on acoustic measures of voice quality (Walzak et al., 2008). In addition, there are no studies of which we are aware that have compared the relative amounts of singing training and acting experience on the acoustics or perception of the singing voice. This is peculiar given the popularity of opera and musical theater, which often require both singing and acting experience. Amongst vocalists with a high level of acting experience, there may be a reprioritization of vocal goals toward emotional genuineness over pitch accuracy or voice quality. In contrast, vocalists with more years of singing training may instead prioritize pitch accuracy and voice quality. In this paper we sought to examine the relationship between acting experience and singing training on the acoustics and perception of the male singing voice.

Pitch accuracy may be considered one of the most salient perceptual dimensions on which we rate the quality of the singing voice. In a national survey of singing pedagogues, intonation, the ability to sing in tune, was regarded as the most important factor in assessing singing talent (Watts et al., 2003). Trained singers are able to reproduce known melodies with a high degree of pitch accuracy, varying between 30 to 42 cents on average (Larrouy-Maestri et al., 2013). Pitch accuracy in the general population has received considerable interest within the last 10 years (for a review, see Hutchins and Peretz, 2012). Although untrained singers can be quite accurate in terms of pitch when singing familiar and unfamiliar tunes (Dalla Bella et al., 2007; Pfordresher et al., 2010), they fare worse than trained singers when producing single pitches; deviating on average by 1.3 semitones from the target pitch compared to 0.5 semitones for trained singers (Ternstrom et al., 1988; Amir et al., 2003; Hutchins and Peretz, 2012). Non-musicians have also been characterized as being “imprecise,” as their fundamental frequency (*F*_0_) for a given pitch can vary across repeated productions (Pfordresher et al., 2010). Thus, the effect of singing training on pitch accuracy appears to depend on the musical context; that is, melodies vs. single pitches.

Where inaccurate pitch production occurs is likely to vary with the structure of the melody. One likely candidate though is the first note of the melody. In a study of untrained child vocalists and trained adult singers, Howard and Angus (1997) found that children were most inaccurate in the pitch of the first note of the melody. In the present study we also examine pitch measures of the first note. How pitch inaccuracy is quantified is an important methodological decision. During vocalization, the rapid opening and closing of the glottis produces a dynamic *F*_0_ contour that varies over time (Fujisaki, 1983). While mean *F*_0_ is often reported, this measure does not capture the range of vocalized *F*_0_. In this study we examine the mean, minimum (floor), and maximum (ceiling) *F*_0_ of the first note in an effort to capture the true range of pitch accuracy. What causes inaccurate pitch production is not fully understood, though it is thought that issues related to voice training, such as poor air support, vocal tension, lack of energy, and poor voice placement are determining factors and that pitch accuracy improves through singing training (Telfer, 1995; Willis and Kenny, 2008). However it remains unclear whether other forms of artistic experience, specifically acting experience, have an effect on singing pitch accuracy. One phenomenon in which acting experience may play a role is through the reprioritization of pitch accuracy during *phrasing*.

In musical theater, phrasing has been described as “the singer’s personal stamp on the song,” where “one performer may sing the lyric with absolute fidelity to the song as written, singing it pitch for pitch, ⋯ while another singer may absolutely transform the same song through her variations” (Deer and Dal Vera, 2008, p. 226). Taylor (2012, p. 34) writes that “performers are not completely circumscribed by the musical text in the meanings and emotions they communicate, as intonation, dynamic range and pitch are relative concepts that are stylistically interpreted.” Thus, phrasing has been suggested to include changes to the intonation, intensity, and pitch from that of the notated score, with the effect of tailoring the meaning and emotions communicated to the individual desires of the singer. As vocalists gain greater acting experience, they may work to refine or emphasize their individuality, which may lead to an increase in deviations from the notated score. Thus, vocalists with a high level of acting experience may deviate more from the notated score than vocalists with less acting experience. Where in the melody these intentional deviations may occur is unknown. However, the first note of the melody is again a likely candidate, as any such deviation at this point would be particularly salient to the listener and may set up expectations about the quality or nature of the ensuing performance.

Artistic phrasing may encompass a broader range of perturbations than pitch and intensity, and include factors related to the perception of “voice quality.” Two acoustic measures that are thought to index the perception of voice quality are jitter (Juslin and Laukka, 2001) and harmonics-to-noise ratio (HNR). The set of acoustic measures thought to capture vocal quality is debated (Raphael et al., 2011). Other perceptual qualities, such as harshness, tenseness, and creakiness have also been implicated in affecting voice quality (Gobl and Nì, 2003). Jitter refers to fine-scale perturbations in *F*_0_ caused by variations in the glottal pressure cycle (Lieberman, 1961; Scherer, 1989). HNR is a measure of the amount of noise in phonation, and refers to the ratio of energy contained at harmonics of *F*_0_ compared to energy that is not (noise; Yumoto et al., 1982). Jitter and HNR are used to assess vocal pathology, with older and pathologically “rough” voices characterized by higher jitter and lower HNR values (Wilcox and Horii, 1980; Ferrand, 2002). HNR has also been associated with the perception of vocal attractiveness (Bruckert et al., 2010). Our investigation examined these spectral features in male vocalists. Previous research suggests that the presence or absence of the “singer’s formant,” a characteristic peak near 3 kHz in the vocal energy spectrum, varies across genders and may be absent in higher female voices (Bartholomew, 1934; Sundberg, 1974; Weiss et al., 2001). As these differences may have added additional variance to our spectral measures, our investigation focused on male vocalists. We operationalize phrasing as deviations from the notated score (e.g., *F*_0_ accuracy, intonation), as well as spectral perturbations of the voice that relate to voice quality.

How a performer’s use of phrasing may affect the perception of the singing voice is unknown, though one candidate is emotional genuineness (Krumhuber and Kappas, 2005; Langner et al., 2010; Scherer et al., 2013). Genuineness refers to the degree to which a listener or observer thinks or feels the vocalist’s expression is a truthful reflection of the vocalist’s physiological, mental, and emotional state. This quality is of particular importance to actors, who use the pejorative term *indicating* to refer to a non-truthful performance. Katselas (2008, p. 109) writes that “to indicate is to show, I repeat, *show* the audience emotion, character through external means ⋯ without really feeling or experiencing the moment. It’s a token, a symbol, an indication, the shell of the thing without internal connection or actual experience.” We hypothesize that vocalists with greater acting experience may sacrifice accurate singing production and voice quality, as measured through increased *F*_0_ deviations, more jitter, and a lower HNR, to achieve greater levels of emotional genuineness.

In this paper we report two experiments that examined the relationships between the relative amounts of acting and singing experience on the acoustics and perception of the singing voice. The first experiment involved acoustical analyses of short phrases that were sung with different emotions and intensities. We expected that vocalists with more years of acting experience would show decreased pitch accuracy, with an *F*_0_ (mean, floor, ceiling) further from the target note pitch, and lower voice quality (increased jitter, lower HNR), relative to vocalists with fewer years of acting training. We also expected that vocalists with more years of singing training would exhibit increased pitch accuracy, with an *F*_0_ (mean, floor, ceiling) closer to the target note pitch, and potentially higher voice quality (higher average HNR, decreased jitter), relative to vocalists with fewer years of singing training. The second experiment examined listeners’ perception of emotional genuineness from vocalist’s singing performances. Listeners rated the emotional genuineness of recordings that were used in Experiment 1. We expected that vocalists with more years of acting experience would be rated as more emotionally genuine, and that these ratings would be associated with increased *F*_0_ deviations, more jitter, and a lower HNR.

In both experiments we examined these relationships using repeated measures linear mixed models (LMMs). This form of analysis is particularly suited to a repeated measures design where covariates are of interest, as the use of repeated measures in traditional multiple regression violates the assumption of independence (Bland and Altman, 1994). LMMs also offer advantages over linear regression and analyses of covariance, allowing for the specification of random intercepts, with the fitting leading to independent intercepts for each vocalist or listener.

## EXPERIMENT 1

Participants were required to sing short statements with five different emotional intentions (calm, happy, sad, angry, and fearful) and two intensities (normal, strong) while having their vocal productions recorded. We predicted that vocalists with more years of acting experience would produce a less pitch-accurate performance, have a lower HNR and more jitter – indicative of lower voice quality – relative to vocalists with fewer years of acting experience. We also predicted that more highly trained singers, as indexed by their years of singing lessons, would produce a more pitch-accurate performance, a higher HNR, and less jitter – indicative of higher voice quality – relative to vocalists with fewer years of singing training. We selected years of acting experience over acting lessons, as actors’ primary form of training in our sample was through active drama performance.

### METHOD

#### Participants

Twelve male vocalists (mean age = 26.3, SD = 3.8) with varying amounts of private or group singing lessons (*M* = 4.8, SD = 3.7), and varying levels of acting experience (*M* = 10.8, SD = 4.0), were recruited from the Toronto acting community. A correlation of vocalists’ years of singing lessons with their years of acting experience was not significant *r*(10) = 0.07, *p* = 0.84, indicating there was no relationship between extent of training in the two domains of interest. Normality of the data were also confirmed with Shapiro–Wilk tests on age (*p* > 0.05), years of acting experience (*p* > 0.05), and years of singing lessons (*p* > 0.05). Participants were native English speakers, and were paid $50 CAD for their participation.

#### Stimuli and apparatus

Two neutral English statements were used (“Kids are talking by the door,” “Dogs are sitting by the door”). Statements were seven syllables in length and were matched in word frequency and familiarity using the MRC psycholinguistic database (Coltheart, 1981). Two isochronous melodies were used; one for the positively valenced emotions, calm and happy (F3, F3, A3, A3, F3, E3, F3), and one for the negatively valenced emotions, sad, angry, and fearful (F3, F3, A^b^3, A^b^3, F3, E3, F3). Both melodies used piano MIDI tones of fixed acoustic intensity, consisting of six eighth notes (300 ms) and ending with a quarter note (600 ms), and were encoded at 16 bit/48 kHz (wav format). Positively and negatively valenced melodies were in the major and minor modes respectively (Dalla Bella et al., 2001).

The stimulus timeline consisted of three main epochs: Task presentation (4500 ms), Count-in (2400 ms), and Vocalization (4800 ms). In the task presentation epoch, the statement and emotion to be produced by the vocalist were presented on screen as text for 4500 ms. Once the text had been on screen for 1000 ms, the melody to be used by the vocalist was sounded (2400 ms). The count-in epoch presented a visual count-in timer (“1,” “2,” “3,” “4”) at an IOI of 600 ms. The start of the vocalize epoch was signaled with a green circle that was displayed for 2400 ms. The stimulus timeline was preceded by an auditory beep (500 ms) and 1000 ms of silence, and ended with an auditory beep (500 ms). Temporal accuracy of the presentation software was confirmed with the Black Box Toolkit (Plant et al., 2004).

Stimuli were presented visually on a 15 inch Macbook Pro running Windows XP SP3 and auditorily over KRK Rocket 5 speakers, controlled by Matlab, 2009b and the Psychophysics Toolbox (3.0.8 SVN 1648, Brainard, 1997). Recordings were performed in a sound-attenuated recording studio equipped with sound baffles. Vocal output was recorded with an AKG C414 B-XLS cardioid microphone with a pop filter, positioned 30 cm from the vocalist, and digitized on a Mac Pro computer with Pro Tools at 16 bit/48 kHz, and a Digidesign 003 mixing workstation.

#### Design and procedure

The experimental design was a 5 (Emotion: calm, happy, sad, angry, fearful) × 2 (Statement: kids, dogs) × 2 (Intensity: normal, strong) × 2 (Repetition) within-subjects design, with 40 trials per participant. A dialog script was used with vocalists. Each emotion was described, along with a vignette describing a scenario involving that emotion. Trials were blocked by emotion. Two presentations orders of emotion were used, and counterbalanced across participants (calm, happy, sad, angry fearful, or sad, angry, fearful, calm, happy). Within emotion blocks, trials were blocked by statement and counterbalanced across participants. For all vocalists, strong intensity productions followed normal intensity productions. An intensity factor was included to capture a broader range of emotional expression (Diener et al., 1985; Sonnemans and Frijda, 1994), which has been shown to affect the acoustics of vocal emotional productions (Banse and Scherer, 1996; Juslin and Laukka, 2001). It was emphasized that vocalists were to produce genuine expressions of emotion, and that they were to prepare themselves physiologically using method acting or emotional memory techniques so as to induce the desired emotion prior to recording. Time was provided between each emotion to allow vocalists to reach the intended emotional state. This form of induction procedure has been used previously in the creation of emotional stimuli (Bänziger et al., 2012). The concept of indicating was also explained, and vocalists were instructed not to produce an indicated performance. Vocalists were told to sing the basic notated pitches, but that they were free to vary acoustic characteristics in order to convey the desired emotion in a genuine manner. Vocalists were standing during all productions. Vocalists were allowed to repeat a given trial until they were comfortable with their production. The final two productions were used in subsequent analyses.

#### Analyses

Recordings were edited using Adobe Audition CS6. Vocal intensity was peak-normalized within each vocalist to retain acoustic intensity variability across the emotions. Recording levels were adjusted across vocalists to prevent clipping, given the range in vocal intensity across participants^1^. Acoustic recordings were analyzed with Praat (Boersma and Weenink, 2013). Fundamental frequency (*F*_0_ mean, floor, and ceiling), HNR, and jitter (local) were extracted^2^. To assess pitch accuracy, *F*_0_ of the first note of the melody was examined (*M*_duration_ = 225.3 ms, SD = 85.35 ms). Three measures of pitch accuracy in the first note were examined: *F*_0_ mean is the average pitch of the first note; *F*_0_ floor is the minimum pitch value during the first note, while *F*_0_ ceiling is the maximum pitch value during the first note. Pitch contours of the first note were converted to cents to provide a normalized measure of inaccuracy from the intended pitch (F3 = 174.614 Hz); a value of 0 cents would indicate perfect accuracy (174.614 Hz), 100 cents would indicate a sharp performance of 1 semitone above the target pitch (184.997 Hz), and -100 cents would indicate a flat performance of 1 semitone below the target pitch (164.814 Hz). Note onsets and offsets were marked in Praat with respect to characteristic changes in the spectrogram, acoustic intensity, and pitch contours. Ten percent of the samples were checked by a second rater (mean inter-rater boundary time difference = 2.1 ms, SD = 2.2 ms). HNR and jitter measures were taken across the voiced portions of the entire utterance.

#### Statistical analyses

Linear mixed models were fitted using the MIXED function in SPSS 22.0. In Experiment 1, all models were fitted with a diagonal covariance structure for the repeated covariance type, which is the default structure for repeated measures in SPSS 22.0. In Experiment 1, analogous models were also fitted using AR(1) and ARH(1), more suited to longitudinal repeated measures, and the more conservative unstructured covariance matrix (Field, 2009). Models fitted with AR(1) and ARH(1) yielded poorer fits, while models fitted with unstructured covariance could not be assessed as the number of parameters to be fitted exceeded the number of observations. Random effects were fitted with a variance components (VC) covariance structure, as is suggested for random intercept models (Field, 2009). All other statistical tests were carried out in Matlab, 2013b or SPSS 22.0.

### RESULTS

Separate repeated measures LMMs were conducted to assess how vocal experience predicted acoustic measures of the singing voice. Five acoustic measures were examined: *F*_0_ (mean, floor, and ceiling), Jitter, and HNR. Repeated measures LMMs were used as each vocalist was recorded singing 40 times, with Vocalist (12) entered as a random effect (intercept), and Emotion (5 levels), Intensity (2), Statement (2), Repetition (2), Singing Lessons (continuous), and Acting Experience (continuous) entered as fixed effects. LMMs were built using a “step-up” strategy, starting with an unconditional means model with only intercepts for fixed and random effects, and then adding in random coefficients (Singer, 1998; Snijders and Bosker, 1999; Raudenbush and Bryk, 2002; Twisk, 2006). For each step, changes to the model fit were assessed with likelihood tests using maximum likelihood (ML) estimation (Twisk, 2006). Factors which significantly improved the model fit were retained. Adding the effect of Repetition (all *p*-values > 0.236), or any of its interactions with Emotion, Intensity, and Statement (all *p*-values > 0.163) were not found to significantly improve model fits for any acoustic parameter and was not included in the final model. Similarly, the interaction of Statement × Intensity did not significantly improve model fits for any acoustic parameter and was not included in the final model (all *p*-values > 0.395). While Statement × Emotion only improved the model fit for *F*_0_ (ceiling), the interaction was retained to facilitate comparisons between models (Cheng et al., 2009).

**FIGURE 1:**
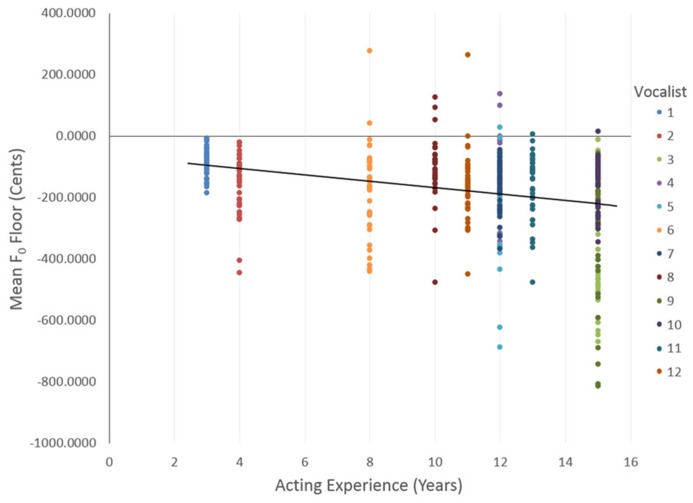
**Vocalists’ years of acting experience and *F*_0_ floor of their first note for all trials.** Solid black line indicates a line of best fit, with a linear regression solution: *F*_0_ floor = -62.66 - 10.37 × Acting experience.

Outcomes for the final models are described in **Table 1**. For *F*_0_ (floor), main effects were reported for Statement, Emotion, and Intensity, indicating that vocalists varied their minimum *F*_0_ depending on their emotional intent or statement. Pairwise comparisons with Bonferroni correction confirmed that Calm (*M* = -229.21, SE = 15.66) exhibited a lower *F*_0_ floor than Happy (*M* = -161.41, SE = 11.81), Angry (*M* = -164.19, SE = 12.44), and Fearful (*M* = -124.85, SE = 15.66), but not Sad (*M* = -184.21, SE = 15.66). Normal intensity emotions (*M* = -191.54, SE = 9.98) had a lower *F*_0_ floor than strong intensity emotions (*M* = -154.17, SE = 10.12). Importantly, vocal experience was found to have a significant effect on vocalists’ *F*_0_ floor, where vocalists with more years of acting experience exhibited a lower *F*_0_ floor, *b* = -9.21, *t*(8.84) = -4.15, *p* = 0.003; illustrated in **Figure 1**. Conversely, vocalists with more years of singing training exhibited a higher *F*_0_ floor in their first note, *b* = 6.40, *t*(8.84) = 2.64, *p* = 0.027. To further examine these effects, we took median splits based on years of Acting Experience: *F*_0 Floor-ActingLow_ = -145.42 cents, SD = 112.34 (*N* = 8), and *F*_0 Floor-ActingHigh_ = -234.0 cents, SD = 170.94 (*N* = 4), and on years of Singing Lessons: *F*_0 Floor__-S__ingingLow_ = -209.8 cents, SD = 152.92 (*N* = 6) and *F*_0 Floor-SingingHigh_ = -140.1 cents, SD = 118.17 (*N* = 6). These results suggest that vocalists with greater acting experience, and vocalists with less singing training, exhibited an *F*_0_ floor that was further from the target pitch. The relationship between the categorical fixed factors and *F*_0_ floor, when controlling for vocal experience, showed significant variance in the intercepts across vocalists var(*u*_0j_) = 2686.33, χ^2^(1) = 52.21, *p* < 0.01.

**Table 1:** Summary of results from linear mixed models in Experiment 1 comparing the effects of vocal experience on acoustic parameters of the voice.

Acoustic parameter	Fixed effects							Random effects
	Continuous		Categorical					Intercept
	Acting	Singing	Statement	Emotion	Intensity	S × E	E× I	Vocalist
*F*_0_ Floor	*F*_(1,8.84)_ = 17.22,	*F*_(1,8.84)_ = 6.98,	*F*_(1,323.28)_ = 10.48,	*F*_(4,139.46)_ = 12.26,	*F*_(1,302.34)_ = 14.75,	*F*_(4,145.77)_ = 0.97,	*F*_(4,138.68)_ = 2.56,	var(*u*_0j_) = 2686.33,
	***p* = 0.003**	***p* = 0.027**	***p* = 0.001**	***p* < 0.001**	***p* < 0.001**	*p* = 0.424	***p* = 0.041**	χ^2^(1) = 52.21 ***p* < 0.01**
*F*_0_ Mean	*F*_(1,10.47)_ = 8.59,	*F*_(1,10.47)_ = 1.08,	*F*_(1,209.1)_ = 9.96,	*F*_(4,86.48)_ = 34.6,	*F*_(1,182.9)_ = 83.26,	*F*_(4,85.21)_ = 0.37,	*F*_(4,86.98)_ = 10.59,	var(*u*_0j_) = 734.63,
	***p* = 0.014**	*p* = 0.321	***p* = 0.002**	***p* < 0.001**	***p* < 0.001**	*p* = 0.828	***p* < 0.001**	χ^2^(1) = 54.21 ***p* < 0.01**
*F*_0_ Ceiling	*F*_(1,10.47)_ = 0.67,	*F*_(1,10.47)_ = 0.09,	*F*_(1,214.76)_ = 9.96,	*F*_(4,116.29)_ = 40.27,	*F*_(1,216.51)_ = 62.63,	*F*_(4,111.53)_ = 2.437,	*F*_(4,86.98)_ = 9.2,	var(*u*_0j_) = 2583.64,
	*p* = 0.43	*p* = 0.776	*p* = 0.303	***p* < 0.001**	***p* < 0.001**	*p* = 0.051	***p* < 0.001**	χ^2^(1) = 47.77 ***p* < 0.01**
Jitter	*F*_(1,11.61)_ = 5.0,	*F*_(1,11.61)_ = 0.033,	*F*_(1,245.44)_ = 20.18,	*F*_(4,99.53)_ = 82.01,	*F*_(1,217.39)_ = 80.03,	*F*_(4,98.179)_ = 2.38,	*F*_(4,86.98)_ = 10.59,	var(*u*_0j_) = 3.61 × 10^-^^6^,
	***p* = 0.046**	*p* = 0.86	***p* < 0.001**	***p* < 0.001**	***p* < 0.001**	*p* = 0.057	***p* < 0.001**	χ^2^(1) = 91.68 ***p* < 0.01**
HNR	*F*_(1,11.80)_ = 2.29,	*F*_(1,11.80)_ = 0.172,	*F*_(1,360.63)_ = 37.28,	*F*_(4,122.16)_ = 294.62,	*F*_(1,339.71)_ = 141.46,	*F*_(4,112.73)_ = 0.369,	*F*_(4,124.91)_ = 26.29,	var(*u*_0j_) = 2.17,
	*p* = 0.156	*p* = 0.686	***p* < 0.001**	***p* < 0.001**	***P* < 0.001**	*p* = 0.83	***p* < 0.001**	χ^2^(1) = 126.64 ***p* < 0.01**

*The significance of the fixed effects was assessed with Type III SS *F*-tests on the final multivariate model. Changes in model fit for fixed effects were assessed with ML estimation. Variance estimates for random effects are reported using REML estimation (Twisk, 2006). Statistically significant *p*-values are highlighted with bold typeface. Fixed effects and interactions that did not significantly improve model fits in any of the acoustic parameters were not included in the final models.*

For *F*_0_ mean, main effects were reported for Statement, Emotion, and Intensity, indicating that vocalists also varied their mean *F*_0_ depending on their emotional intent or statement. Pairwise comparisons with Bonferroni correction confirmed that Calm (*M* = -46.36, SE = 7.15) exhibited a lower *F*_0_ mean than Happy (*M* = -6.2, SE = 7.56), Sad (*M* = -23.26, SE = 7.94), Angry (*M* = 35.71, SE = 10.6), and Fearful (*M* = 25.59, SE = 9.92). Normal intensity emotions (*M* = -28.16, SE = 6.72) also had a lower *F*_0_ mean than strong intensity emotions (*M* = 22.35, SE = 7.82). 

Importantly, acting experience was found to have a significant effect on vocalists’ *F*_0_ mean, where vocalists with more years of acting experience exhibited a lower mean *F*_0_, *b* = -4.92, *t*(10.47) = -2.93, *p* = 0.014. To further examine these pitch differences, we took median splits on Acting Experience: *F*_0 Mean-ActingLow_ = 10.79 cents, SD = 90.52 (*N* = 8) and *F*_0 Mean-ActingHigh_ = -21.7 cents, SD = 82.69 (*N* = 4). These results suggest that vocalists with more years of acting experience were more flat on the first note. The mean absolute pitch of the first note across all vocalists was 55.2 cents (SD = 70.06). These results suggest that vocalists in general sang the first note of the melody within half a semitone of the target pitch. The relationship between the categorical fixed factors and *F*_0_ mean, when controlling for vocal experience, also showed significant variance in the intercepts across vocalists, var(*u*_0j_) = 734.63, χ^2^(1) = 54.21, *p* < 0.01.

For *F*_0_ ceiling, main effects were reported for Emotion and Intensity, indicating that vocalists varied their *F*_0_ ceiling depending on their emotional intent. Pairwise comparisons with Bonferroni correction confirmed that Calm (*M* = 75.78, SE = 13.46) had a lower *F*_0_ ceiling than Happy (*M* = 142.48, SE = 14.72), Sad (*M* = -153.0, SE = 18.52), Angry (*M* = 216.2, SE = 21.47), and Fearful (*M* = 237.75, SE = 19.62). Normal intensity emotions (*M* = 119.28, SE = 13.74) also had a lower *F*_0_ ceiling than strong intensity emotions (*M* = 210.80, SE = 15.59). No relationship was reported between vocal experience and *F*_0_ ceiling. The relationship between the categorical fixed factors and *F*_0_ ceiling also showed significant variance in the intercepts across vocalists, var(*u*_0j_) = 2583.64, χ^2^(1) = 47.77, *p* < 0.01.

For Jitter, main effects were reported for Statement, Emotion, and Intensity, indicating that the level of jitter in vocalists’ voices varied depending on their emotional intent or statement. Pairwise comparisons with Bonferroni correction confirmed that Calm (*M* = 0.011, SE = 4.21 × 10^-^^4^) had less jitter than Happy (*M* = 0.014, SE = 4.43 × 10^-^^4^), Sad (*M* = 0.013, SE = 4.57 × 10^-^^4^), Angry (*M* = 0.017, SE = 5.46 × 10^-^^4^), and Fearful (*M* = 0.017, SE = 5.62 × 10^-^^4^). Normal intensity emotions (*M* = 0.013, SE = 4.09 × 10^-^^4^) had less jitter than strong intensity emotions (*M* = 0.015 SE = 4.47 × 10^-^^4^). These findings are important as they demonstrate that the level of jitter in a vocalist’s voice can be affected by both lexical and emotional goals. Following from this, Acting Experience was found to have a significant effect on vocalists’ jitter levels, where vocalists with more years of acting experience exhibited a higher level of vocal jitter, *b* = 2.31 × 10^-^^4^, *t*(11.61) = 2.24, *p* = 0.046. To further examine this effect, we took median splits based on years of Acting Experience: Jitter _ActingLow_ = 1.37% × 10^-^^2^%, SD = 5.0 × 10^-^^3^ (*N* = 8), and Jitter _ActingHigh_ = 1.49% × 10^-^^2^%, SD = 4.1 × 10^-^^2^ (*N* = 4). These results suggest that vocalists with more years of acting experience had higher levels of vocal jitter. No relationship was reported between jitter and years of Singing lessons. The relationship between our fixed factors and jitter also showed significant variance in the intercepts across vocalists, var(*u*_0j_) = 3.61 × 10^-^^6^, χ^2^(1) = 91.68, *p* < 0.01.

For HNR, main effects were reported for Statement, Emotion, and Intensity, indicating that vocalists varied the HNR in their voice depending on their emotional intent or statement. Pairwise comparisons with Bonferroni correction confirmed that Calm (*M* = 18.58, SE = 0.37) had a higher HNR than Happy (*M* = 15.99, SE = 0.38), Sad (*M* = 17.41, SE = 0.38), Angry (*M* = 13.0, SE = 0.38), and Fearful (*M* = 14.19, SE = 0.37). Normal intensity emotions (*M* = 16.57, SE = 0.36) also had a higher HNR than strong intensity emotions (*M* = -15.1, SE = 0.36). As with jitter, this is an important finding as it confirms that the HNR in a vocalist’s voice is not fixed. No relationships were found between HNR and Acting experience, and HNR and Singing lessons. The relationship between the fixed factors and HNR was also found to show significant variance in the intercepts across vocalists, var(*u*_0j_) = 2.17, χ^2^(1) = 126.64, *p* < 0.01.

### DISCUSSION

The results of Experiment 1 confirmed that different types of vocal training, in the form of years of acting experience and years of singing lessons, produced differences in the acoustics of the singing voice. Vocalists with more years of acting experience exhibited a lower *F*_0_ floor, with the most experienced actors singing on average up to 234 cents below the target pitch, a deviation of more than 2 semitones (E^b^3 instead of F3). In contrast, vocalists with more years of singing lessons exhibited a *F*_0_ floor that was closer to the target pitch relative to less trained singers. Vocalists with more years of acting experience also sang the first note flat, with a lower *F*_0_ mean relative to vocalists with fewer years of acting experience. Overall, vocalists’ mean pitch for the first note varied within half a semitone of the target pitch. No relationships were reported for *F*_0_ ceiling and vocal training. On measures of voice quality, vocalists with more years of acting experience exhibited higher levels of jitter. No relationship was found between vocal experience and HNR. Importantly, both jitter and HNR varied consistently across emotion, intensity, and statement, confirming that like emotional speech (Dupuis and Pichora-Fuller, in press), these spectral aspects of the emotional singing voice are not fixed within a vocalist. These results partially support our hypothesis, and suggest that vocalists with more years of acting experience sung with a lower voice quality, as indexed by greater pitch inaccuracy and higher levels of jitter. No effects were reported between singing training and measures of voice quality, and so our hypotheses regarding these acoustic measures was not supported. Significant random intercepts were reported in all acoustic features, indicating a consistent tendency by some vocalists to exhibit higher or lower levels of these acoustic measures than other vocalists, even when controlling for the effects of their vocal experience background. These results support the use of LMMs in the analysis of Experiment 1, by accounting for additional variance within acoustic parameters across the vocalists.

Collectively, these results suggest that the type and amount of vocal training a singer receives may have a significant effect on acoustic measures of their singing voice. In particular, vocalists with more years of acting experience sung with a lower voice quality and greater pitch inaccuracy. We theorize that such deviations may have been intentional so as to increase the perception of emotional genuineness during their performances. To assess this relationship we conducted a second experiment in which listeners’ evaluated the emotional genuineness of vocalists’ singing performances.

## EXPERIMENT 2

Experiment 2 examined listeners’ perception of emotional genuineness from vocalists’ singing recordings. In Experiment 1, vocalists with more years of acting experience exhibited increased pitch inaccuracy and higher levels of vocal jitter. We theorized these deviations were an intentional singing technique by more experienced actors to increase the genuineness of their performances. We hypothesized that vocalists with more years of acting experience would be rated by listeners as possessing higher levels of emotional genuineness. We further expected that acoustic measures of the voice would also be associated with listeners’ perception of genuineness. We hypothesized that recordings with a lower *F*_0_ floor and increased jitter would be rated as more genuine. While no effect was reported between vocal training and HNR in Experiment 1, based on our original theoretical predictions we hypothesized that recordings with a lower HNR would be rated as more emotionally genuine.

### METHOD

#### Participants

Fourteen adults (7 female, mean age = 29.29, SD = 7.49) were recruited from the Ryerson university community. The experiment took approximately 30 min. No participant from Experiment 1 took part in Experiment 2.

#### Stimulus and apparatus

A subset of acoustic recordings from Experiment 1 were used as stimuli in Experiment 2. Ten recordings were used for each vocalist, one for each emotional category and emotional intensity level. The statement used was “Kids are talking by the door.” Stimuli were presented acoustically with a Macbook Pro laptop and Logitech X-140 powered external speakers.

#### Design and procedure

The experimental design was a 12 (Vocalist) × 5 (Emotion: calm, happy, sad, angry, fearful) × 2 (Intensity: normal, strong) within-subjects design, with 120 trials per participant. Trials were presented in random order. On each trial, participants were asked to rate the genuineness of the vocalist’s production using a 5-point scale (1 = not at all genuine to 5 = very genuine). Prior to the experiment, the concept of emotional genuineness was explained to participants as follows: “Emotional genuineness concerns whether you believe that the vocalist was truly experiencing the emotion they were portraying. Emotional genuineness should not be confused with the intensity or clarity of the portrayed emotion.” Loudness was adjusted to a comfortable level, and was held constant across presentations.

#### Analyses

The relationships between listeners’ genuineness ratings and vocalists’ years of acting experience and singing lessons were assessed with LMMs. The statistical procedures described in Experiment 1 were reused in Experiment 2. As in Experiment 1, analogous models were fitted using AR(1) and ARH(1), and the more conservative unstructured covariance matrix (Field, 2009). Models fitted with AR(1) and ARH(1) yielded poorer fits, while models fitted with unstructured covariance failed to converge. Random effects were again fitted with a VC covariance structure.

### RESULTS

A three-level repeated measures LMM was conducted to assess how vocal experience predicted listeners’ ratings of emotional genuineness. A repeated measures LMM was used as each vocalist was presented 10 times to each of the 14 listeners. Vocalist (12) was entered as a random effect, and was further added as a random effect nested within Listener (14). The variables Emotion (5 levels), Intensity (2), Singing Lessons (continuous), and Acting Experience (continuous) were entered as fixed effects. Based on Experiment 1 results, we did not expect Singing lessons to have a significant effect on listeners’ ratings of genuineness. However for completeness, its effect on the model was examined. Singing Lessons did not significantly improve the model fit (*p* = 0.542), and was not included in the final model.

**FIGURE 2:**
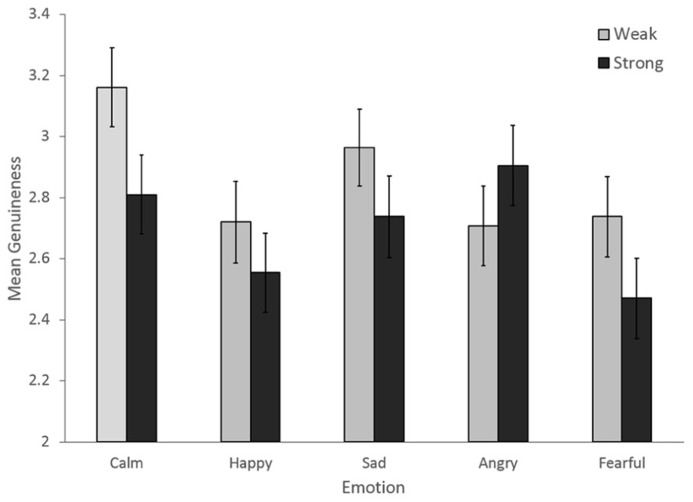
**Mean genuineness ratings showing the Emotion by Intensity interaction in Experiment 2.** Error bars denote the standard error of the means.

Outcomes of the final model are described in **Table 2**. Main effects were reported for Emotion and Intensity, as was an interaction between Emotion and Intensity, illustrated in **Figure 2**. Pairwise comparisons with Bonferroni correction confirmed that Calm (*M* = 2.985, SE = 0.12) was rated as significantly more genuine than Happy (*M* = 2.637, SE = 0.12) and Fearful (*M* = 2.604, SE = 0.12), but not Angry (*M* = 2.807, SE = 0.12) or Sad (*M* = 2.851, SE = 0.12). Less intense emotions (*M* = 2.858, SE = 0.12) were also rated as more genuine than more intense emotions (*M* = 2.695, SE = 0.12). Less intense emotions were rated as more genuine for all emotions except angry, suggesting a role in the interaction.

**Table 2:** Summary of results from the linear mixed model in Experiment 2 comparing listeners’ ratings of emotional genuineness with vocalist training background of the vocalist.

Perceptual parameter	Fixed effects				Random effects
	Continuous	Categorical			Intercepts	
	Acting	Emotion	Intensity	E × I	Listener	Listener × Vocalist
Genuineness	*F*_(1,151.44)_ = 20.03,	*F*_(4,574.23)_ = 9.44,	*F*_(1,1498.97)_ = 12.53,	*F*_(4,574.23)_ = 4.22,	var(*u*_0j_) = 0.172,	var(*u*_0j_) = 0.066,
	***p* < 0.001**	***p* < 0.001**	***p* < 0.001**	***p* = 0.002**	χ^2^(1) = 203.27, ***p* < 0.01**	χ^2^(1) = 31.47, ***p* < 0.01**

*The significance of the fixed effects was assessed with Type III SS *F*-tests on the final multivariate model. Changes in model fit for fixed effects were assessed with ML estimation. Variance estimates for random effects are reported using REML estimation (Twisk, 2006). Statistically significant p-values are highlighted with bold typeface. Fixed effects that did not significantly improve the model fit were not included in the final model.*

**FIGURE 3:**
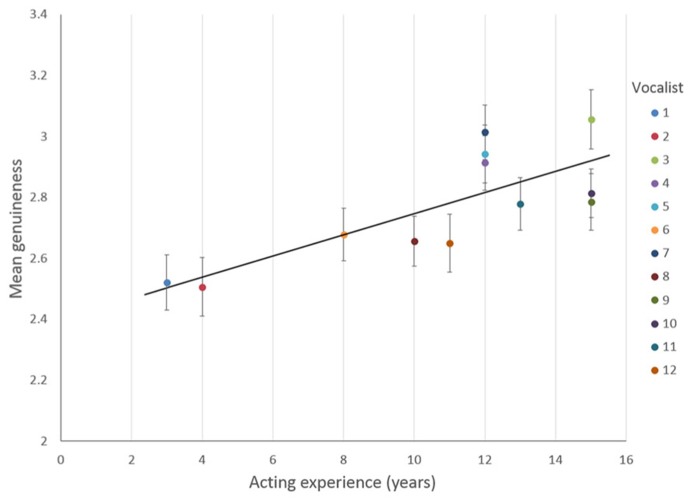
**Listeners’ mean genuineness ratings for each vocalist, and vocalists’ years of acting experience in Experiment 2**. Solid black line indicates a line of best fit, with a linear regression solution: Genuineness = 2.4 + 0.035 × Acting experience. Error bars denote the standard error of the means.

Importantly, vocalists’ acting experience was found to have a significant effect on listeners’ ratings of emotional genuineness, where vocalists with more years of acting experience were rated as more emotionally genuine, *b* = 0.035, *t*(150.45) = 4.46, *p* < 0.001, illustrated in **Figure 3**. This result supports our main hypothesis that vocalists with more years of acting experience would be rated as more emotionally genuine. The relationship between the categorical fixed factors and Genuineness, when controlling for acting experience, showed significant variance in the intercepts across Listener var(*u*_0j_) = 0.172, χ^2^(1) = 203.27, *p* < 0.01, and in the intercepts across Vocalist within Listener, var(*u*_0j_) = 0.066, χ^2^(1) = 31.47, *p* < 0.01.

To determine if a relationship existed between the acoustic features examined in Experiment 1 and listeners’ ratings of emotional genuineness, we ran a LMM with Emotion (5 levels), Intensity (2), *F*_0_ Floor (continuous), *F*_0_ Mean (continuous), *F*_0_ Ceiling (continuous), *F*_0_ Jitter (continuous), and HNR (continuous) entered as fixed effects. Adding the effects of *F*_0_ mean (*p* = 0.94) and *F*_0_ ceiling (*p* = 0.258) were not found to significantly improve model fits for emotional genuineness, and were not included in the final model. The main effect of Intensity was significant until the addition of the final acoustic parameter HNR, after which it was no longer significant (*p* = 0.386). To facilitate a comparison with previous models, this effect was retained in the final model.

**FIGURE 4:**
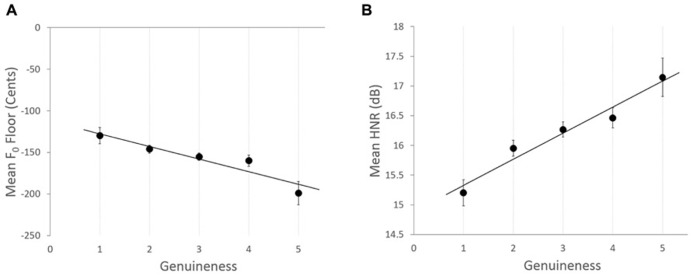
**Relationships between vocalists’ acoustic features and in Experiment 2 for (A) Mean *F*_0_ floor for each genuineness rating category**. Solid black line indicates a line of best fit, with a linear regression solution: *F*_0_ floor = -112.49 - 15.21 × Genuineness. **(B)** Mean HNR for each genuineness rating category in Experiment 2. Solid black line indicates a line of best fit, with a linear regression solution: HNR = 14.89 + 0.44× Genuineness. For both figures, error bars denote the standard error of the means.

Outcomes of the final model are described in **Table 3**. Main effects were reported for Emotion as was an interaction between Emotion and Intensity. Importantly, three of the five acoustic parameters examined were found to affect listeners’ ratings of emotional genuineness. Recordings with a lower *F*_0_ floor were rated as more emotionally genuine, *b* = -5.97 × 10^-^^4^, *t*(1125.03) = -2.75, *p* = 0.006, illustrated in **Figure 4A**. Recordings with increased jitter were also rated as more emotionally genuine, *b* = 16.93, *t*(950.72) = 2.05, *p* = 0.041. Finally, recordings with increased HNR were also rated as more emotionally genuine, *b* = 0.095, *t*(932.06) = 5.02, *p* < 0.001, illustrated in **Figure 4B**. The model continued to show significant variance in the intercepts across Listener var(*u*_0j_) = 0.170, χ^2^(1) = 203.27, *p* < 0.01, and in the intercepts across Vocalist within Listener, var(*u*_0j_) = 0.080, χ^2^(1) = 31.47, *p* < 0.01.

**Table 3:** Summary of results from the linear mixed model in Experiment 2 comparing listener ratings of emotional genuineness with acoustic measures of the voice.

Perceptual parameter	Fixed effects						Random effects
	Continuous			Categorical			Intercepts	
	*F_0_* (floor)	Jitter	HNR	Emotion	Intensity	E × I	Listener	Listener × Vocalist
Genuineness	*F*_(1,151.44)_ = 7.56,	*F*_(4,574.23)_ = 4.19,	*F*_(1,1498.97)_ = 25.25,	*F*_(4,778.63)_ = 7.38,	*F*_(1,1610.45)_ = 0.75,	*F*_(4,700.78)_ = 6.69,	var(*u*_0j_) = 0.170,	var(*u*_0j_) = 0.080,
	*** p* = 0.006**	***p* = 0.041**	***p*<0.001**	***p*<0.001**	* p* = 0.39	***p*<0.001**	χ^2^(1) = 203.27, ***p*<0.01**	χ^2^(1) = 31.47, ***p*<0.01**

*The significance of the fixed effects was assessed with Type III SS *F*-tests on the final multivariate model. Changes in model fit for fixed effects were assessed with ML estimation. Variance estimates for random effects are reported using REML estimation (Twisk, 2006). Statistically significant *p*-values are highlighted with bold typeface. Fixed effects that did not significantly improve the model fit and were not included in the previous model (***Table 2***), were not included in the final model.*

### DISCUSSION

The results of Experiment 2 confirmed that listeners’ ratings of emotional genuineness were related to the level of acting experience of the vocalist, and to the acoustic features of the voice for: *F*_0_ floor, Jitter, and HNR. Vocalists with more years of acting experience were rated as more emotionally genuine relative to vocalists with fewer years of acting experience, supporting our main hypothesis. No relationship was reported between years of singing lessons and emotional genuineness, as was expected based on findings from Experiment 1. The experimental factors Emotion and Intensity were also both found to affect listeners’ perception of genuineness. Calm productions were overall rated as the most genuine, while fearful productions were rated as the least genuine. Interestingly, less intense emotions were rated as more genuine than strongly intense emotions. This suggests that vocalists’ emotional displays were more believable when their expressions were less intense. However, the interaction between emotion intensity suggested that while this was the case for most emotions, strongly intense anger appeared to be rated as more genuine than less intense anger. Significant random intercepts were also reported for ratings of genuineness, both for individual listeners and for vocalists within listeners, indicating a consistent tendency by listeners to rate the genuineness of recordings more or less between one another, and for some vocalists over others. These results support the use of LMMs in the analysis of Experiment 2, by accounting for additional variance within genuineness ratings across listeners.

Importantly, three of the five acoustic measures examined were found to be significantly related to listeners’ ratings of emotional genuineness. Recordings with a lower *F*_0_ floor were rated as more emotionally genuine, as were recordings with increased jitter, both of which supported our hypothesis. HNR was also associated with listeners’ ratings of emotional genuineness. However, counter to our hypothesis, recordings with a higher HNR were rated as more emotionally genuine. Thus, our hypothesis regarding HNR was only partially supported, as while HNR was associated with listeners’ perception of emotion, the direction of the relationship was opposite to our predictions.

It is unclear why recordings with a higher mean HNR were rated as more emotionally genuine. A tentative explanation is that genuineness ratings may have been influenced by factors related to vocal attractiveness. Voices with a higher average HNR tend to be judged as more attractive (Bruckert et al., 2010). Consistent with the “halo effect” (Zebrowitz et al., 1996), participants are more willing to ascribe positive attributes, such as likability, to voices that are judged to be attractive (Zuckerman and Driver, 1989).

## GENERAL DISCUSSION

Two experiments provided converging evidence that different types of vocal training affect the acoustics of the male singing voice in divergent ways, which concomitantly affect listeners’ perception of emotional genuineness. Vocalists’ exhibited differences in their fundamental frequency (*F*_0_ floor, mean), and levels of jitter that were related to their years of vocal experience. Vocalists with more years of acting experience exhibited increased pitch inaccuracy with a lower minimum *F*_0_ and a lower mean *F*_0_ relative to the target pitch of the first note, and increased vocal jitter. In contrast, vocalists with more years of singing training exhibited a higher *F*_0_ floor that was closer to the target pitch (less flat). No relationship was found between vocal training and HNR. Collectively, these results suggested that vocalists with more years of acting experience sung with a lower voice quality. It was theorized that vocalists’ reduction in voice quality was an intentional phrasing technique – particularly amongst vocalists with a lot of acting experience – to increase the perception of their emotional genuineness. Findings from the perceptual experiment supported this hypothesis. Vocalists with more years of acting experience were rated as more genuine. No relationship was found between the amount of singing training and the perception of genuineness. As hypothesized, recordings with a lower *F*_0_ floor and increased vocal jitter were rated as more emotionally genuine. As hypothesized, HNR was also associated with listeners’ perception of genuineness, however the direction of the effect went against our expectations as voices containing a higher HNR were rated as more genuine. This latter finding may reflect a moderating role of vocal attractiveness in judgments of emotional genuineness (Zuckerman and Driver, 1989; Bruckert et al., 2010). Overall, these findings support our two main hypotheses that different types of vocal training affect the acoustics of the male singing voice in unique ways, which in turn affect listeners’ perception of emotional genuineness.

An important outcome of this investigation was the identification of acoustic measures that affected listeners’ perception of emotional genuineness. All three acoustic features varied consistently with the emotional category and intensity of the vocalist, confirming that the spectral qualities jitter and HNR of the voice are not fixed for a given vocalist. While *F*_0_ is generally under the conscious control of the vocalist, it is unclear whether the same is true of jitter or HNR. Thus an interesting avenue for future research would be to examine if vocalists can be trained to consciously control the levels of jitter and HNR in the voice. These outcomes would be relevant to vocal pedagogy in those performers seeking to increase their emotional genuineness with listeners. The findings would also be relevant to vocal attractiveness research, where increased HNR is thought to influence the perception of vocal attractiveness (Bruckert et al., 2010).

The results of the present study indicated that vocalists with more years of acting experience sung with a lower voice quality. We theorized that these performers were seeking to put their “personal stamp on the song” (Deer and Dal Vera, 2008, p. 226), where the use of stylistic deviations may function to enhance the individual uniqueness or emotionality of the performance. The connection between stylistic deviations and performer uniqueness has been reported previously. Repp (1992) examined the expressive timing deviations of 24 international concert pianists in their performances of Schubert’s Träumerei. While all pianists exhibited characteristic tempo changes matching the structure of the work, large individual differences were reported in which performers deviated extensively from the expected timing curve, and particularly for two of the more famous performers. In a follow-up study involving graduate piano students’ performances of the same work, Repp (1995) found that the students also exhibited similar timing patterns, but that their deviations were much more homogeneous than those of the concert pianists. These findings suggest that *phrasing*, as it is referred to in the acting world, may be a general artistic phenomenon in which more experienced performers seek to differentiate themselves with their own unique style. Thus, while phrasing may involve the deviation or degradation of a typical performance, it may be done so purposefully and should not be considered erroneous. We believe that the acoustic deviations exhibited by vocalists with a large amount of acting experience in this study should be viewed in this light. In the present study these relationships were examined using performers who had varying levels of singing and acting experience. In the future these effects could be examined more directly with participants who were more closely matched on their years of singing and acting experience.

The importance of genuineness in emotion research has received increasing attention over the last decade. Differences in the production and perception of genuine versus simulated emotions is a topic of intense debate (Russell et al., 2003; Scherer, 2003; Vogt and André, 2005). The use of induction procedures is also gaining use amongst researchers who require ecologically valid stimuli. (Douglas-Cowie et al., 2007; Bänziger et al., 2012). In this study an induction procedure was used in an attempt to induce the physiological and mental correlates of the emotion being expressed. Likewise, researchers are increasingly assessing observers’ beliefs about the genuineness of their stimuli (Langner et al., 2010). The results of the present study suggest that vocal training type and the duration of experience may serve as useful predictors of a vocalist’s emotional genuineness, and that these factors should be considered in future genuineness studies.

## CONCLUSION

The goals of a vocal performer are varied and many: accurate pitch reproduction, desired voice quality, clear intelligibility, precise timing, and intended emotional inflection. The findings of the present study confirm that these factors are not independent, and that performers may prioritize different aspects of their performance due to differences in their vocal training. These acoustic changes have important consequences on listeners’ evaluation of emotion, and highlight the nuanced quality of individual differences in singing performance.

## Conflict of Interest Statement

The authors declare that the research was conducted in the absence of any commercial or financial relationships that could be construed as a potential conflict of interest.

## Footnotes

1 Prior to recording, participants were asked to sing and speak several test sentences with a very angry emotional intention. Very angry was selected as this emotion was often the loudest during the audition pre-screening sessions. Recording levels were adjusted based on the loudest of these test productions. For occasional instances of clipping or “popping” during the recording sessions, the actor was asked to repeat the trial.

2 Pitch contour was extracted with an autocorrelation algorithm (ac) in Praat, with the following settings: pitch floor 70 Hz, pitch ceiling 420 Hz, very accurate pitch contour tracking, maximum periodicity candidates 15, silence threshold 0.08, voicing threshold 0.45, octave cost 0.01, octave-jump cost 0.4, voiced/unvoiced cost 0.14, time step 0.004. Jitter (local) was extracted using a periodic cross-correlation algorithm in Praat (periodic, cc).
